# The LifeWatch approach to the exploration of distributed species information

**DOI:** 10.3897/zookeys.463.8397

**Published:** 2014-12-12

**Authors:** Daniel Fuentes, Nicola Fiore

**Affiliations:** 1 Estación Biológica de Doñana, Consejo Superior de Investigaciones Científicas (CSIC), Spain.; 2 Ecology Unit, University of Salento, Italy

**Keywords:** Online taxonomic resources, interoperable web services, information retrieval, taxonomy

## Abstract

This paper introduces a new method of automatically extracting, integrating and presenting information regarding species from the most relevant online taxonomic resources. First, the information is extracted and joined using data wrappers and integration solutions. Then, an analytical tool is used to provide a visual representation of the data. The information is then integrated into a user friendly content management system. The proposal has been implemented using data from the Global Biodiversity Information Facility (GBIF), the Catalogue of Life (CoL), the World Register of Marine Species (WoRMS), the Integrated Taxonomic Information System (ITIS) and the Global Names Index (GNI). The approach improves data quality, avoiding taxonomic and nomenclature errors whilst increasing the availability and accessibility of the information.

## Introduction

Detailed information of species can be queried by the scientific community through multiple online taxonomic resources which are accessible on the Web. An online taxonomic resource is a megascience platform that harvests, processes and provides biodiversity data of animals, plants, fungi and micro-organisms. The information included in these portals describes taxonomies, synonyms, references, images and distributions, etc. The online taxonomic resources are a aggregation (or mash-up) designed to collate the data of all organisms set in the context of a taxonomic hierarchy and of their distribution.

In general, a comprehensive online taxonomic resource cover information on all kinds of organisms like in Catalogue of Life (CoL, http://www.catalogueoflife.org), Discover Life (http://www.discoverlife.org/), Encyclopedia of Life (EoL, http://eol.org), Global Biodiversity Information Facility (GBIF, http://www.gbif.org), Biodiversity Heritage Library (BHL, http://www.biodiversitylibrary.org), the Integrated Taxonomic Information System (ITIS, http://www.itis.gov) and the Global Names Index (GNI, http://gni.globalnames.org). However, it can also focuses on a limited area of biodiversity, such as World Register of Marine Species (WoRMS, http://www.marinespecies.org), International Nucleotide Sequence Database Collaboration (INSDC, http://www.insdc.org) and JSTOR Plant Science (http://plants.jstor.org).

It is estimated that the number of species on the planet that have been documented by scientists has risen to 1.9 million (Chapman 2009). Taxonomists have been tasked with cataloguing and quantifying the Earth’s biodiversity. Their progress is measured in code-compliant descriptions that include text, images, type material and molecular sequences (Hardisty et al. 2013). These experts often disagree about the best classification for a given group of organisms, and there is no universal taxonomy (Patterson et al. 2014, David et al. 2012). However, the taxonomic name has remained as the most commonly shared identifier that spans sequences, specimens, and publications despite the wealth of possible connections between biodiversity data objects (Patterson et al. 2010).

As we move towards a digital data world, we are increasingly reliant on the internet as a source of information (Thessen et al. 2011, Patterson 2014). Unfortunately, at present it is often tedious, even with the help of new technologies, to obtain information on a taxonomic name, either to track its origins and subsequent use, or to verify that it has been correctly used. Taxonomists have to consult primary literature because they consider that online resources are incomplete (Thessen et al. 2012, Franz et al. 2008).

Nowadays, species identification errors come from diverse causes: the variation in data quality and cross-linkages between databases, an inadequate updating of information and the lack of a single authoritative world taxonomic resource for the definition of the taxa cause. Moreover, the taxonomy itself is subject to continuous evolution, since the elements that it classifies continue to evolve. Hence, taxonomic resources will differ in their composition even if they claim to be comprehensive. They will change as new knowledge develops due to scientific and technological advances in the field of software development and evolution (Franz and Thau 2011, Franz et al. 2008).

Online taxonomic resources reflect these differences by supporting several scientific classifications resulting in mismatched records and inflated species numbers. Divergences in names and taxonomies are frequently found due to consulted databases are fed from disparate sources. The consequence of misspelled names and bad taxonomy is erroneous to scientific conclusions (De Broyer et al. 2011, Katsanevakis et al. 2013, Parr et al. 2012).

Contextually, the maintenance and management of information uploaded, error avoidance and the resolution of inconsistencies combined with the data control of treatments, make species list management a great effort (Zachos 2013). Thus, a strong case is presented, for the integration and harmonization of the existing information distributed regarding species classifications. The lack of tools facilitating this task becomes a fundamental obstacle.

The LifeWatch research e-infrastructure (http://www.lifewatch.eu) does not try to compose its own taxonomy from different sources. LifeWatch has come to an agreement with the different sources of taxonomic backbone information so as to be able to offer their usage in the LifeWatch framework, for example, in order to disambiguate the species names in queries. In addition, LifeWatch is working closely with all initiatives in the domain including GBIF, the Global Names Architecture (GNA, http://www.globalnames.org), EoL, CoL, Pan-European Species directories Infrastructure (PESI, http://www.eu-nomen.eu), and national authorities, to provide the taxonomic capabilities needed for its purposes (Giddy et al. 2009).

This paper presents a method that facilitates the exploration of existing species information from distributed sources, through a set of interoperable web services. It enables links to the most important on-line biodiversity databases to retrieve information about taxonomy, synonyms, common names, etc. The rest of this paper is structured as follows: In section 2 a brief description of the infrastructure where the presented service is placed. Section 3 outlines the most widely used online taxonomic resources and software tools. The design and implementation of our proposal are presented in Section 4 and the last section contains some concluding remarks.

## LifeWatch in a nutshell

**Figure 1.**
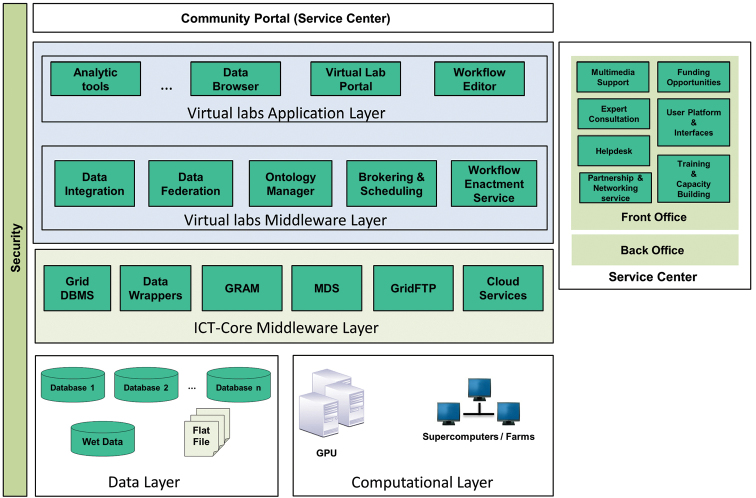
LifeWatch architecture in layers.

LifeWatch is a European research e-infrastructure (ESFRI, http://ec.europa.eu/research/infrastructures/index_en.cfm?pg=esfri) project for biodiversity science and ecosystems research (now entering its construction phase) that collaborates with scientists and engineers from across the European Union. Figure 1 shows the LifeWatch architecture divided in layers, including a range of new services and tools to help researchers communicate, share data, analyze results, create models, manage projects and organize training.

Services can be put together in three main groups:

- *Core ICT support*: The LifeWatch ICT Infrastructure is a system of distributed nodes distribution system that provides access to and processing of biodiversity data from a variety of sources through common open interfaces (Giddy et al. 2009). In this context, flexible and durable solutions for storage, computing, networking and hosting are included providing services with the technology support required.
- *Virtual Laboratories*: To provide researchers with a common point of access and share the data, the LifeWatch infrastructure includes a set of virtual labs. A virtual lab is an interoperable computing environment that allows researchers to update the database and to use analytical tools to extract specific information from the data. Furthermore, it supports multidisciplinary international collaboration between researchers working in different time zones.
- *Community support*: Due to the diverse range of available tools, the community support element of the platform brings people and expertise together. Thus, providing access to all services, and assisting participating organizations and scientists with training programmes, technical advice, grant information and other resources.

The LifeWatch functional requirements concern the types of operations that the users need in order to find, access, process and view data. They include:

- Searching and browsing mechanisms for distributed data and services.
- Uniform identity framework for data and services.
- Access to existing data and services, distributed among multiple organisations. Data and service providers continue to manage their data (and services) independently as now, including control of the creation and modification of data/services. However, data can be accessed by authorised users located anywhere through a generic mechanism defined by LifeWatch.
- Mechanisms for source data preservation, such as the access to past versions of data sets that have been used to produce secondary information.
- Capture data from users and lightweight devices, including field sensors and networks providing continuous streams of new data, and portable computing devices, often with intermittent connectivity.
- Mechanisms for data analysis as well as mapping and modelling tools, using standard ways to manipulate and view data.
- Mechanisms for data fusion, integrating different sources (such as sensor data, biodiversity parameters, geographic data, primary data, workflow execution), to allow fast retrieval at different levels of detail, for example, in analysis and visualisation.
- Support the understanding of results by the user, by providing tools and mechanisms to enhance knowledge extraction from discovery as well as from analysis results.

## State of art

In the rest of the paper, we will consider the following concepts related to taxonomy taking into account the list of terms used in GBIF (Hawksworth 2010):

**Species:** A taxon at the rank.

**Classification.** Hierarchical system in which items may be grouped, with little or no ancillary data.

**Checklist.** List of names within a limited context.

**Treatment.** Description of a taxon

**Aggregation.** The drawing together of digital biodiversity data from multiple sources.

**Scientific name.** The scientific name of a taxon at any rank above the species group consists of one name, that of a species of two names (binomen), and that of a subspecies three names (a trinomen).

**Accepted name.** the designation adopted by an author as the correct name for a taxon under consideration.

**Valid name.** of an available name, one acceptable under the provisions of the taxonomic resource and which is the correct name for a taxon in an author’s taxonomic judgement.

**Synonym.** Each of two or more names of the same rank used to denote the same taxon.

### The scope of megascience platforms processing biodiversity information

Online taxonomic resources provide information about the taxonomic status of a taxon as well as synonymous relations, they facilitate the taxonomic data capture, help input data control processes and integrate information in other databases and infrastructures (Triebel et al. 2012). Despite almost all these taxonomic resources containing information about all living things, each has its own data domains, providers, scope of contents and user communities. For instance: occurrences and records in GBIF, taxonomic checklists and classifications in CoL, names in the Global Names Usage Bank (GNUB, http://www.globalnames.org/GNUB), taxonomy in marine species in WoRMS, zoological publications and nomenclatures in The Official Registry of Zoological Nomenclature (ZooBank, http://zoobank.org), knowledge data and multimedia objects in EoL, DNA barcoding sequences in International Barcode of Life (iBOL, http://ibol.org), etc.

Scientific names and taxonomies are of essential interest in major biodiversity platforms (Boyle et al. 2013, Metzger et al. 2013, Patterson et al. 2010) and are typically fed by individual scientists and institutions researching data. However, they may alternatively be supplied by primary data collected from other databases (Nozères et al. 2012) which creates a graph, in which taxonomic resources are the nodes and data flows the connectors, creating dependences between them. For example, EoL contains information from several taxonomic hierarchies like GBIF, GNA, iBOL, International Nucleotide Sequence Database Collaboration (INSDC), JSTOR Plant Science and Biodiversity Heritage Library (BHL), The International Plant Names Index (IPNI, http://www.ipni.org), etc.

In the future, data flows will be even more complicated due to the growth in the number of initiatives, infrastructures and collaborations that cover taxonomy and classification challenges. The new biology based on the big data world is envisaged as a discipline with a strong data-centric character and a growing role for informatics. The responsibility for managing data from many sources will probably carried out by modules or nodes that serve specified subdisciplines. The nodes will aggregate heterogeneous content within a particular subdomain, making it discoverable and available to end users (Patterson 2014, Thesen and Patterson 2011). An example that follows this model is the Global Names Architecture, which index, organize and interconnect on-line information about organisms and their names.

### Interoperability solutions

Today, one of the main challenges in bioinformatics is the implementation of the interoperability in an environment where interdisciplinary cooperation is key to scientific understanding (Berendsohn et al. 2011, Fielding 2000, Remsen et al. 2006. Data sharing is essential to fostering the collaboration and large-scale analysis needed for the successful treatment of the initiatives connected with biodiversity (Costello et al. 2013, Hine et al. 2003). Currently, there are two main proposals to achieve the interoperability desired across such systems and data; federating and brokering. In the federating solution (Heimbigner et al. 1985), the interoperability is achieved by a set of service buses based on the SOA architecture binding the user and the provider. Initiatives that follow this solution are based on federal specifications, covering data and metadata models, predefined vocabularies and ontologies, service interfaces and binding protocols. The Infrastructure for Spatial Information in the Europe (INSPIRE, http://inspire.ec.europa.eu) directive or the National Spatial Data Infrastructure (http://www.fgdc.gov/nsdi/nsdi.html) are two examples of such a federated approach.

However, in the brokering approach (Nativi et al. 2011) the heterogeneity is addressed by focusing on mediation rather than standardization. It proposes a User-Broker-Producer model by which the Broker consists of multiple support components facilitating the discovery of, semantic and natural language mediation, data access services, workflow process, and publishing. The brokering model has been adopted by the European approach to Global Earth Observation System of Systems (EuroGEOSS, http://www.eurogeoss.eu) project. Following the functional requirements, the brokering option is selected as all data providers use a different specification to interoperate. Therefore, a middleware (composed of brokers) interconnect clients and online taxonomic resources in a common infrastructure.

### Tools

In the last years, some tools have already been developed for the exploration of distributed information about taxonomies. New advances in taxonomic publication processes are designed to speed information automatically to diverse users. One method dealt with a solution for special citation of taxonomic work when used in wiki pages by combining both the original non-wiki source and the respective wiki page (Penev et al. 2011). However, another approach sets out a workflow that describes the assembly of elements from a Scratchpad taxon page (http://scratchpads.eu) to export a structured XML file (Blagoderov et al. 2010). Methods of semantic tagging and semantic enhancements, text and data processing, publishing and dissemination in taxonomy have provided an increase in open access literature and journals aiming at rapid publication of taxonomic treatments, including new publication models such as semantically enhanced information (Penev et al. 2010). Also, the software package DELTA includes a data-basing program which stores morphological data for export in different forms and acts as a manager of taxonomic research (Coleman et al. 2010).

Recently, many organizations have developed different software tools to harvest, publish and share data (Katsanevakis et al. 2012, Kennedy et al. 2006, Wu et al. 2009). GBIF offers community tools to enable the integration of biodiversity data from heterogeneous sources using standards and protocols; an example is the Integrated Publishing Toolkit (IPT, http://ipt.gbif.org) which enables the publication of content in databases or text files using open standards Darwin Core (DwC, http://rs.tdwg.org/dwc) and the Ecological Metadata Language (EML, http://knb.ecoinformatics.org/software/eml/). The GBIF Spreadsheet Processor is a similar tool intended for smaller occurrence datasets stored in excel files. The limited number of concepts in Darwin Core offer a strength in usability but is weak for observational descriptions (Hill et al. 2010). The Biological Collection Access Service for Europe (BioCASE, http://www.biocase.org) has developed BioCASE Provider Software (BPS, http://www.biocase.org/products/provider_software), a middleware that facilitates the connection and mapping of data into XML schemes such as the Access to Biological Collections Data standard (ABCD, http://www.tdwg.org/standards/115/download/ABCD_v206.html). The main advantage of ABCD is the large set of concepts available relative to a species observation. This however, can be challenging to use owing to concept ambiguity. Biodiversity Information Standards (TDWG) is the organisation that works on defining standards in the field of biodiversity informatics. The most widely deployed formats for occurrence data are DwC and ABCD. The TCS (Taxonomic Concept Transfer Schema) was also developed for exchange taxonomic data but it defines only the taxonomic backbone.

## Taxonomic information retrieval tool

This section presents a tool that explores taxonomic information from online taxonomic resources. Given the name of one species, this approach links to different sources to showing taxonomic information and other related data. The design has followed the LifeWatch data requirements described in Section 2 for the data access and visualization.

### Design

**Figure 2.**
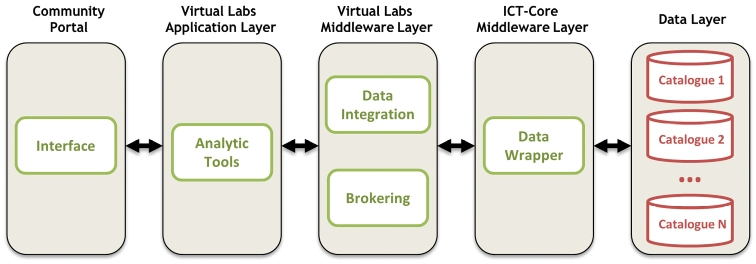
General structure of the service in correspondence with the LifeWatch infrastructure layers.

A graphical representation of the design of the method is presented in Figure 2. It can be noticed that follows the steps of the brokering approach. Layers and modules are shown, which are necessary for the access, extraction, integration, analysis and representation of the data devolving from online taxonomic resources of the LifeWatch architecture (illustrated in Figure 1).

The information is retrieved from data providers and flows through the system from one layer to another. In each layer the information is filtered, selected and formatted to finally facilitate scientists in the analysis of specific species information.

The Data layer contains the distributed taxonomic resources that feed the application with their information. Each resource contains a specification of interoperable services which describe how to access the data.

The ICT-Core Middleware layer includes the Data Wrapper module which accesses the taxonomic resource, queries the information about the requested taxon and extracts the specific fields that the interface shows. Features such as the specification, metadata, request and response are in different resources. Thus, when a new taxonomic resource is added to the system, the module is modified and new ETL (Extract, Transform and Load) solutions are created to obtain the required information.

The Virtual Labs Middleware layer contains two modules. First, once the data from all online taxonomic resources is obtained, the information is joined together in the Data Integration module. This information facilitates the data management and data representation by the broker and the analytic tools respectively.

Second, the Brokering module manages all the data flow in the application using a broker, defining species concepts which are shown to the user. The broker converges disparate vocabularies and enables uniformity of search and access in divergent online taxonomic resources. It receives the name of a taxon from the user interface and calls the Data Integration module to query the taxon in all the taxonomic resources. Subsequently, the result is passed to the next layer for analytical purposes. Finally, the species information generated is sent to the graphical user interface.

The Virtual Labs Application layer includes all the analytical tools to support the visualization of the extracted data from online taxonomic resources using reports, graphs, tabs, rows, colors, etc., improving the exploration and the information driven-decisions.

Lastly, a graphical user interface shows the results with different options to analyze and download the information. The management of the taxonomic resources in the system is flexible, which means that the addition or deletion of a taxonomic resource only supposes the modification of the Data Wrapper module. To incorporate a new online taxonomic resource it is necessary to map the information retrieval from the resource to the specific concepts that the system manages (taxonomies, synonyms, valid and accepted names, etc.). Hence, there is an abstraction layer between the ICT-Core Middleware layer and the Virtual Labs Middleware layer where the implementation details of the taxonomic resources are not relevant to the rest of the design.

### Implementation

Following the previous design a taxonomic tool has been implemented to facilitate the exploration of taxonomies, accepted names and synonyms, using the information from five online taxonomic services; GBIF, CoL and WoRMS, ITIS and GNI. The last resource, GNI, is a compilation of all the various namestrings that have been used as scientific names, whether correctly or not, with variant spellings and mis-spellings. In this sense, GNI cannot be considered a source of taxonomic information as CoL or WoRMS.

**Figure 3.**
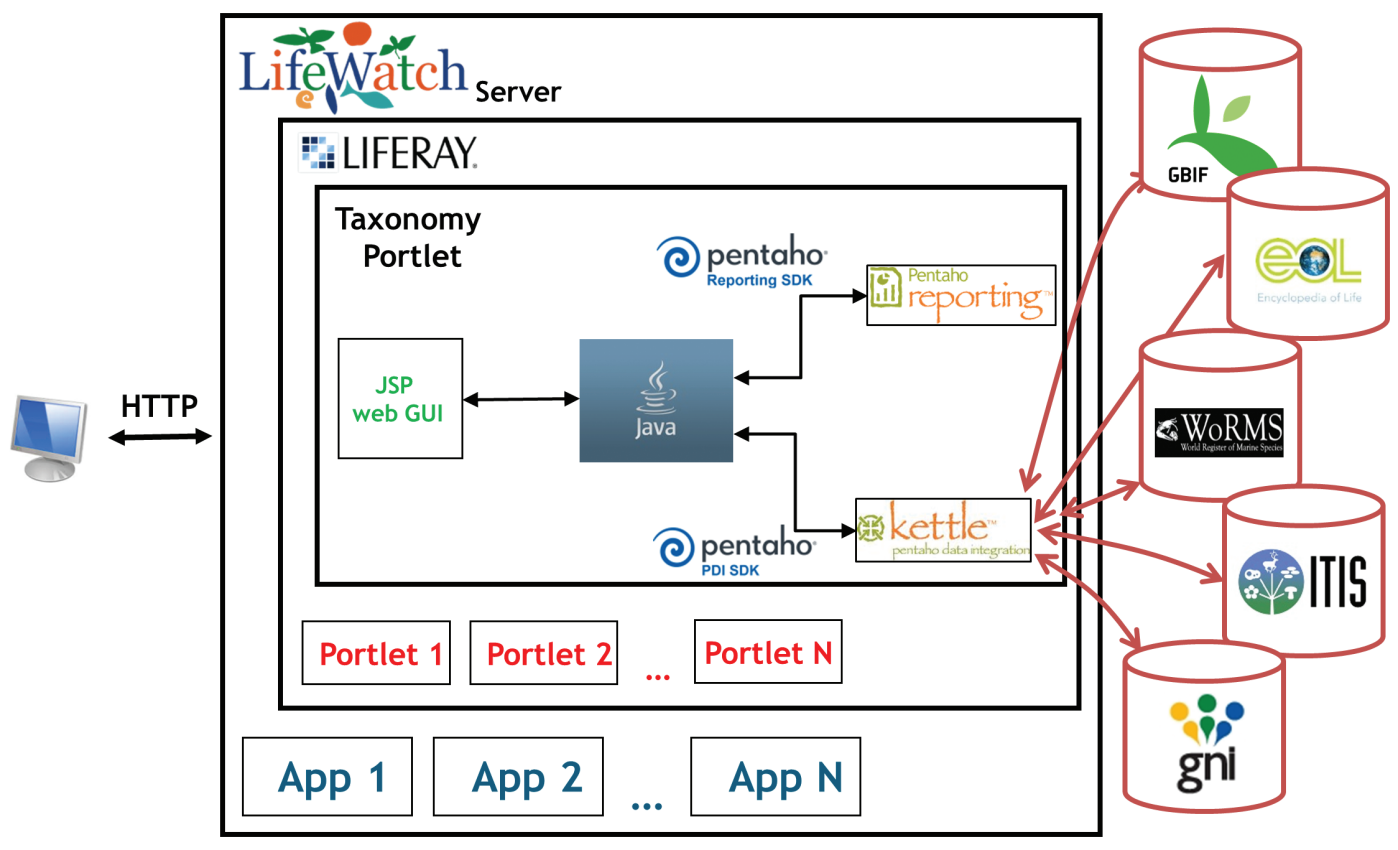
Implementation of the service with the used technologies.

The structure of the solution is represented in Figure 3, where three main elements are distinguished: the online taxonomic resources, the server containing the application that access and shows the information and the user who provides inputting species names.

The server is the principal element in the figure as it provides fundamental infrastructure services. It accommodates the services implemented according to the LifeWatch requirements described in Section 2. These services are listed in four categories: Core Basic Services, Supporting Basic Services, Supporting Thematic Services and Specific Thematic Services. A more detailed list of services and its descriptions can be found in the LifeWatch Reference Model (Giddy et al. 2009). The presented tool is an approximation of the Taxonomy Access Service placed in the Supporting Thematic Services category. An example of such an application is Liferay web portal that contains the infrastructure website which is the service access point. In Liferay, applications are deployed in the form of portlets. We have developed a portlet that implements the search of taxonomic information using the previous design and a business intelligence solution called Pentaho (http://www.pentaho.com).

The three online taxonomic resources make up the current Data layer. They offer interoperable web services and interfaces that facilitate data queries from external applications and integrate such information into other systems. Due to the web services’ specification in any taxonomic resource differs, a new wrapper solution has to be designed to consult each of them. In the portlet, the Data Wrapper module is implemented by the Pentaho Data Integration tool (also known as Kettle). This tool permits the design of transformations, enabling ETL capabilities to form requests, process responses and locate information in each taxonomic resource. Three separate transformations are defined because both ways to query data and the XML structure in responses are different in these taxonomic resources.

The modules contained in the Virtual Labs Middleware Layer are implemented using different software tools. First, the Pentaho Data Integration is again used for the development of the Data Integration module. Once the information of each taxonomic resource is available separately, a new transformation is designed using this tool to join them together in a common XML file.

In the Brokering module, a Java program implements the broker using libraries. The program provides the link between the Analytic tool, the Data Integration, and the Data wrapper modules. When a user introduces the name of a species in the application, the broker sends the request to the Data wrapper module. To do this, it uses the libraries contained in the Pentaho Data Integration SDK to call the transformations which in turn implements the wrappers from the Java program. Subsequently, the Broker sends the information to the Data Integration module. As a result, a XML file contains the data from the three taxonomic resources using common labels.

Finally, the broker sends this file to the Analytic tool, through the libraries defined by the the Pentaho Reporting SDK, which consequently generates a report with the information.

The Analytic tool module is implemented using a report temfig designed by the tool Pentaho Report Design Wizard. This temfig produces a report in which the information is organized using dynamic tables (available in pdf, html and xls). The report is progressed to the graphical interface that illustrating the final results and available for download.

The application also has a data exportation option in compliance with the Darwin Core standard. Darwin Core is an internationally recognized standard for biodiversity data exchange, used by GBIF and other organizations to encode data related to organism names, taxonomies, references, etc. This option provides sufficient flexibility to support specific tasks, allowing advanced users to build custom applications tailored to particular needs (Chapman 2005).

## Results

**Figure 4.**
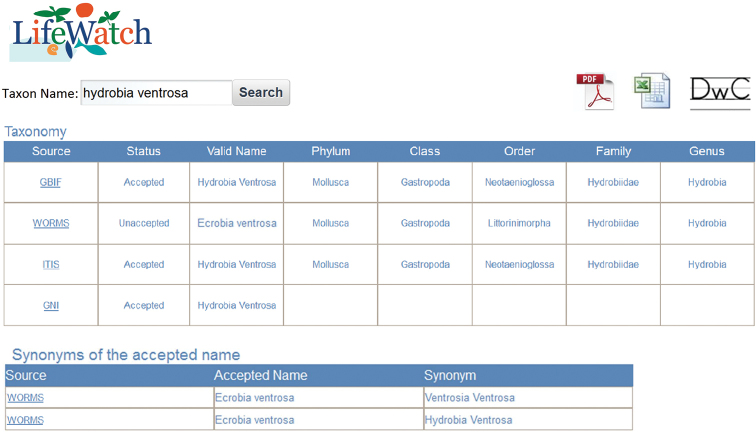
Result of searching *Hydrobia Ventrosa* using our proposal.

Figure 4 shows an example using the proposed implementation. The taxon name introduced by the user is *Hydrobia Ventrosa*, a small brackish water snail. This taxon can be found in GBIF, WoRMS, ITIS and GNI but not in CoL, consequently there are no results for CoL in the application. The web service offered by GNI only permits checking if the provided taxon is a valid name, but the taxonomic information cannot be obtained.

It is evident that GBIF and ITIS show the same taxonomy. However, *Hydrobia Ventrosa* is an accepted name for GBIF, ITIS and GNI but not for WoRMS and the order in WoRMS (*Littorinimorpha*) is also different from the other taxonomic resources (*Neotaenioglossa*). Moreover, given that the accepted name in WoRMS is *Ecrobia Ventrosa*, this taxonomic resource indicates two synonymous names for the same species: *Ventrosia Ventrosa* and (the introduced) *Hydrobia Ventrosa*. Classifications and synonyms of both taxonomic resources appear together in two separated tables which can be downloaded in different formats including, an XML file following the Darwin Core standard. In the first column, the name of the resource shows a direct link to the website in order to obtain more details about the found taxon such as citations, environment, taxonomic history, etc.

**Figure 5.**
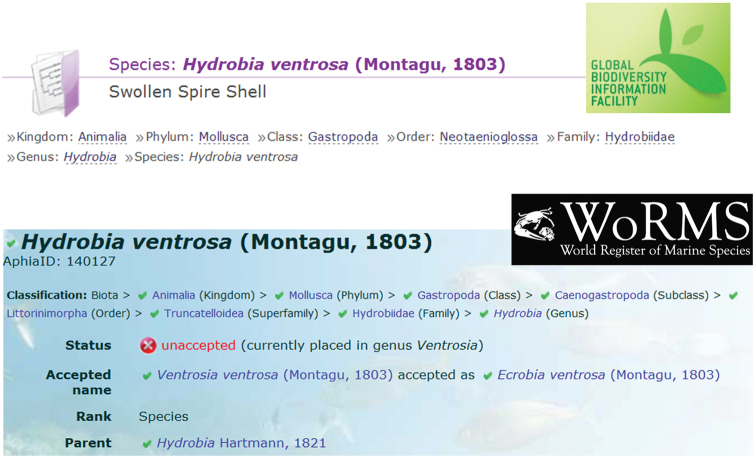
Some details about the results of searching *Hydrobia Ventrosa* in GBIF and WoRMS online taxonomic resources.

Part of the results of a species query in GBIF and WoRMS are represented in Figure 5. From comparisons drawn in both figures, it is clear that our proposal facilitates the visualization of the data in combination with a time effective search that increases availability and accessibility whilst reducing errors in names and classifications.

### Experimentation in a real context

The application is used by some researchers based in the Ecology Unit at the University of Salento (Lecce, Italy). The work is focused on experimental research in aquatic ecosystems. The implemented system has many benefits and enables the reconciliation of species information in different online taxonomic resources.

Firstly, the time that scientists spend researching a taxon has been drastically reduced. Currently, CoL, GBIF, WoRMS, ITIS and GNI all have different websites, interfaces and tools (which the reseacher would need to use). With the proposed system, users don’t need to consider the range of research methods as the search is combined including all online taxonomic resources. Furthermore, the application permits users to introduce a list of taxa showing the results in a crosstab report.

Secondly, we noticed that almost all the scientists base their research on two or more online taxonomic resources. The same taxon cannot appear in a taxonomic resource but can be classified with various synonyms in another. In some cases, especially in old species lists, the same taxon appears with various synonyms provoking confusion. This application permits the resolution of names and synonyms, consequently reducing the size of the lists and avoiding false results and conclusions.

Finally, the method is useful to scientists who work with new or recently-discovered species. In these cases, accepted names and synonyms change frequently. The application helps to find divergences in taxonomies and accepted names between online taxonomic resources.

## Conclusion

A method has been presented to obtain taxonomic information from the main online taxonomic resources. A solution divided in modules has been designed to automatically extract and represent information about taxonomies, synonyms and accepted names. The proposed solution has been used in a real context and a very promising and competitive performance for avoiding errors and false results has been achieved.

## Internet resources

Access to Biological Collections Data (ABCD) – http://www.tdwg.org/standards/115/download/ABCD_v206.html

BioCASE – http://www.biocase.org

BioCASE Provider Software (BPS) – http://www.biocase.org/products/provider_software

Biodiversity Heritage Library (BHL) – http://www.biodiversitylibrary.org

Catalogue of Life (COL) – http://www.catalogueoflife.org

Darwin Core – http://rs.tdwg.org/dwc

Discover Life – http://www.discoverlife.org

Ecological Metadata Language (EML) – http://knb.ecoinformatics.org/software/eml.

Encyclopedia of Life (EOL) – http://eol.org

EuroGEOSS – http://www.eurogeoss.eu

European Strategy Forum on Research Infrastructures (ESFRI) – http://ec.europa.eu/research/infrastructures/index_en.cfm?pg=esfri

Global Biodiversity Information Facility (GBIF) – http://www.gbif.org

Global Names Architecture (GNA) – http://www.globalnames.org

Global Names Index (GNI) – http://gni.globalnames.org

Global Names Usage Bank (GNUB) – http://www.globalnames.org/GNUB

Infrastructure for Spatial Information in the Europe (INSPIRE) – http://inspire.ec.europa.eu

Integrated Taxonomic Information System (ITIS) – http://www.itis.gov

International Barcode of Life (iBOL) – http://ibol.org

International Nucleotide Sequence Database Collaboration (INSDC) – http://www.insdc.org

International Plant Names Index (IPNI) – http://www.ipni.org

Integrated Publishing Toolkit (IPT) – http://ipt.gbif.org

JSTOR Plant Science – http://plants.jstor.org

Liferay – http://www.liferay.com

LifeWatch – http://www.lifewatch.eu

National Spatial Data Infrastructure (NSDI) – http://www.fgdc.gov/nsdi/nsdi.html

Pan–European Species directories Infrastructure (PESI)– http://www.eu-nomen.eu

Pentaho – http://www.pentaho.com

World Register of Marine Species (WoRMS) – http://www.marinespecies.org

Zoological Nomenclature (ZooBank) – http://zoobank.org
